# The particularities and challenges of establishing a curricular teaching unit on interprofessional communication in healthcare

**DOI:** 10.3205/zma001843

**Published:** 2026-04-15

**Authors:** Sabine Fredersdorf, Lars S. Maier, Ilona Stocker, Franziska Staab, Andreas Wiesner, Christine Fehlner, Jobst-Hendrik Schultz, Stephanie Keil

**Affiliations:** 1 University Hospital Regensburg, Clinic and Polyclinic for Internal Medicine II, Regensburg, Germany; 2 University Hospital Regensburg, Nursing Staff Development, Regensburg, Germany; 3 Bavarian Red Cross, Regensburg, Germany; 4 University of Regensburg, Faculty of Medicine, Dean’s Office, Regensburg, Germany; 5 Heidelberg University Hospital, Clinic for General Internal Medicine and Psychosomatics, Heidelberg, Germany

**Keywords:** interprofessional, communication, roles, healthcare

## Abstract

**Background:**

Numerous studies have demonstrated substantial deficits in interprofessional (IP) communication, which contribute to reduced quality of patient care and increased healthcare expenditure. Against this backdrop, a course aiming for strengthening IP competencies was established.

**Methods:**

The target groups were medical students (MS; n=168), nursing trainees (NT; n=30), and paramedic trainees (PT; n=28). The course focused on role clarification and communication and was delivered using simulation-based training scenarios (IP feedback, team communication, and handover). A comprehensive evaluation was performed using a Likert scale (1-6; 1=I agree, 6=I disagree; mean±SD).

**Results:**

In the evaluation, MS reported significantly less interest in the topics than NT (“Topics were interesting to me”: NT 1.9±1.8 vs. MS 3.3±1.8, p<0.05). In contrast to MS and PT, NT perceived the content as well aligned with their level of knowledge (NT 1.3±0.7 vs. MS 3.6±2.0 vs. PT 4.4±1.3, p<0.05). MS rated the time allocated for interprofessional exchange as sufficient, whereas PT rated it as insufficient (MS 1.9±0.3 vs. PT 3.1±2.4, p<0.05). More than 92% of participants rated all training scenarios as clinically relevant. The relevance of the IP topic was praised, while the uneven ratios of the participating professions was criticized.

**Discussion and conclusion:**

The evaluation suggests that interest in IP content varies considerably among participants. Given the high relevance of the topic, this should not result in IP training being offered only to those who are already interested; instead, IP courses should be mandatory and integrated into the curriculum. Specific challenges in establishing IP teaching must be considered, such as ensuring curricular placement aligned with learners’ prior knowledge across professional groups.

## Zusammenfassung

**Hintergrund:**

Zahlreiche Studien belegen ein relevantes Problem in der interprofessionellen (IP) Kommunikation, das zur Verschlechterung der Patient:innenversorgung und Steigerung der Gesundheitskosten führt. Vor diesem Hintergrund wurde ein Kurs zur Förderung IP-Kompetenzen etabliert.

**Methodik:**

Zielgruppe waren Medizinstudierende (MS n=168), Auszubildende der Pflege (PA, n=30) sowie Notfallsanitäter:innen in Ausbildung (NotS, n=28). Der Kurs wurde mit den Schwerpunkten Rollenverständnis und Kommunikation mittels simulationsbasierten Trainingsszenarien (IP-Feedback, Teamkommunikation und Übergabe) durchgeführt und umfassend evaluiert (Likert 1-6, 1=Ich stimme zu, 6=Ich stimme nicht zu; MW±SD).

**Ergebnisse:**

In der Evaluation gaben MS gegenüber den PA signifikant weniger Interesse an den Themen an („Themen für mich interessant“, PA1,9±1,8 vs. MS3,3±1,8, p<0,05). Im Gegensatz zu den MS und NotS empfanden die PA die Inhalte als gut auf ihren Wissensstand abgestimmt (PA1,3±0,7 vs MS 3,6±2,0 vs NotS 4,4±1,3, p<0,05). Den MS empfanden die Zeit für Austausch im Gegensatz zu den NotS als ausreichend (MS1,9±0,3 vs NotS 3,1±2,4, p<0,05). Alle Trainings wurden von den meisten (>92%) als klinisch relevant eingestuft. Gelobt wurde die Relevanz des IP-Themas, kritisiert die ungleichen Zahlenverhältnisse zwischen den Disziplinen.

**Diskussion und Schlussfolgerung:**

Die Evaluation deutet an, dass nicht alle Teilnehmer*innen an IP-Inhalten gleichermaßen interessiert sind. Dies sollte wegen der Themenrelevanz nicht dazu führen, IP-Kurse nur den Interessierten anzubieten, z.B. durch verpflichtende, curricular verankerte Kurse. Bei der Etablierung von IP-Lehre sind spezifische Hürden zu berücksichtigen, wie z.B. die curriculare Platzierung angepasst an das Vorwissen der beteiligten Berufsgruppen.

## Introduction

Experiences from clinical practice, as well as numerous studies, have shown that deficits in adjustment and communication within healthcare teams lead to reduced quality of patient care, increased healthcare costs and decreased workplace satisfaction. Communication failures between different health professions (HP) constitute a frequent cause of medical errors and impaired patient outcomes and contribute substantially to rising healthcare expenditure [1]. An analysis conducted by U.S. governmental authorities (Joint Commission for Hospital Accreditation) attributed 70% of 2,455 documented adverse events to communication errors, 75% of which resulted in patient deaths [2]. IP communication training enhances patient safety by fostering psychological safety [3]; and is further supported by a culture of open error communication, flat hierarchies, and active speaking-up practices, as described by Lee et al. [4]. In a review, Lee et al. demonstrated the relationship between ineffective communication and stressors among surgical teams [5], while Lakin highlighted the IP challenges that arise in the care of critically ill patients [6].

To fulfil future professional roles as Communicators and Collaborators [7], it is essential that learners acquire IP competencies and develop positive attitudes towards other HP [8]. Mutual understanding between professional groups is fundamental [9]. According to the Interprofessional Education Collaborative (IPEC) framework, the core IP competencies comprise four domains that must be strengthened in training programs [10]:

1. Communication
2. Teamwork
3. Values and ethics
4. Roles, responsibilities and accountabilities

The benefits of IP teaching on IP attitudes have been demonstrated in numerous publications. For example, an IP elective course in palliative medicine in Dresden included MS and trainees from various HP (including nursing, geriatric care and physiotherapy) to receive one-on-one support for seminars, observerships and communication trainings [11]. Quantitative analyses showed significant increases in mutual appreciation as well as in understanding roles and competencies. 

Similarly, Liaw et al. demonstrated that simulation-based communication training for the management of critically ill patients led to significant improvements in confidence regarding IP communication and to increased appreciation of IP learning among MS and NT [12]. 

### Assessment of needs

At the Faculty of Medicine at the University of Regensburg, only limited IP teaching currently exists during the Preclinical Basic Science and Clinical Science phases of medical studies. Two IP training wards are available during the final-year clerkships (Practical Year), although only a small proportion of MS gain access to these late in their studies [13]. A voluntary nursing-led practical training program (e.g., urinary catheter placement) exists for both MS and NT.

Interprofessional learning objectives have been incorporated into both the National Competence-Based Catalogue of Learning Objectives for Medicine (NKLM) and the Nursing Professions Act as overarching competencies [14], [15], comprising the four core competencies of the IPEC framework. Ruebling et al. demonstrated that introducing IP courses early in training can sustainably improve attitudes towards other HP [14].

## Project description

The analysis indicated a substantial demand for IP teaching units during the Clinical Science phase. On the initiative of the Dean’s Office, a working group (WG) was established, comprising nursing staff from the Department of Personnel Development (nursing education and training), members of the Dean’s Office, a specialist in internal medicine, MS, and paramedic instructors. This WG developed the structural foundations required to design and implement IP teaching units. 

Within the WG, a sequence of interlinked teaching sessions was developed, based on the IPEC framework, to promote mutual understanding of IP roles and IP communication with a focus on team communication, feedback, and handover. The “teamwork” domain was taught only to competency level 2 according to the NKLM, due to early training stages, cohort size, and time constraints. The “values and ethics” domain was postponed because the WG considered alignment with the participants’ educational and experiential levels insufficient. 

Following an extensive literature review, learning objectives were formulated based on the IPEC framework and NKLM 2.0 ([https://nklm.de/zend/menu], Chapters VIII.2 and VIII.3). Table 1 (Tab. 1) lists the resulting learning objectives according to IPEC [10].

The design and piloting phase lasted ten months and was conducted adapted on Kern’s approach [16]. Subsequently, the teaching units were integrated into the core curricula for MS, NT, PT. 

## Teaching and learning methods

To achieve the interprofessional (IP) learning objectives, various teaching formats were applied, as described in the literature. IP learning has been shown to result in better performance in IP topics compared with monoprofessional courses [17]. A commonly used approach is IP simulation training using mannequins [17] or simulation scenarios [12]. 

**Figure 1:**
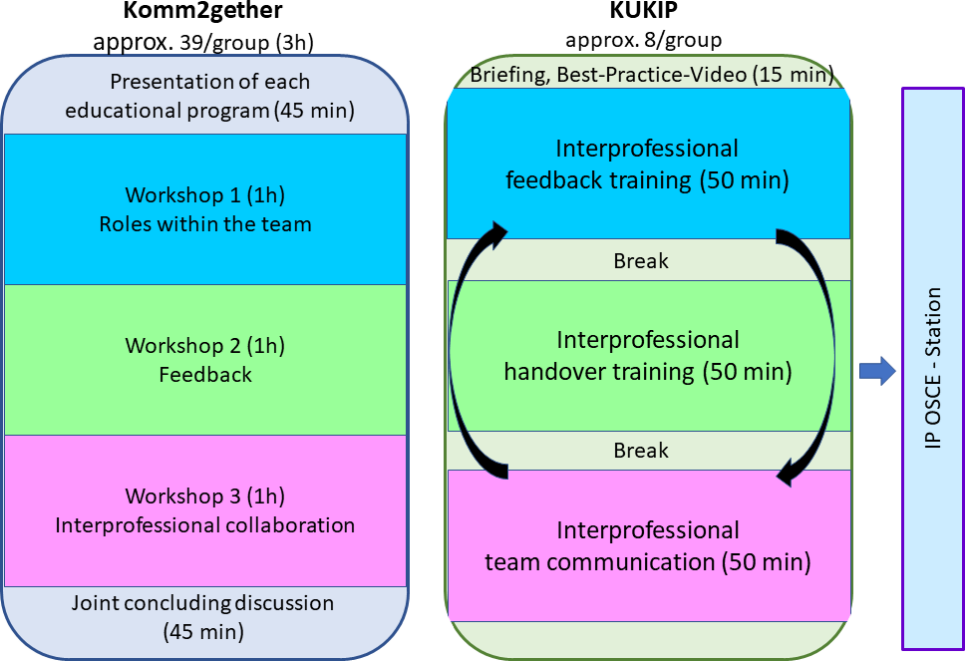
Overview of the 2 interprofessional course units

The IP teaching unit was divided into two sections (see figure 1 (Fig. 1)). In the first part of the teaching unit (Komm2gether), theoretical foundations were established, and participants were sensitized to the topic. In the second part (KUKIP), practical training session in small IP groups were conducted based on the contents previously taught. 

An IP OSCE station was integrated into an established OSCE station circuit for MS to assess learning outcomes.

### Komm2gether

For this part of the course, the total of 226 participants (30 NT, 28 PT, 168 MS) was divided into two large groups and the course was conducted separately. After presenting the relevance of IP communication and introducing the participating HP in the lecture hall, participants were allocated to breakout groups for three 60-minute workshops focusing on team roles, feedback, and IP collaboration.

After short introductory lectures, the topics were discussed using clinical vignettes and corresponding questions in the small IP groups (see table 2 (Tab. 2)). The results of the group work were recorded on flip charts and presented and discussed in a final plenary session in the lecture hall. 

### KUKIP

During the course, three small groups (each with eight participants: five to six MS, two NT or two PT) rotated through three learning stations on IP communication (IP feedback, handover and team communication) to deepen their understanding of the theoretical content of Komm2gether through practical exercises. The group size resulted from the assignment of at least 2 participants from each profession to the small groups. At the beginning, a best-practice video demonstrating good teamwork and clear role understanding was shown. The course was held by pre-trained MS as student tutors, while trained nursing staff as learning facilitators rotated through the groups. Sample vignettes for each station are provided in attachment 1 .

Based on the children's game, in the *telephone game* station, a standardized patient (SP) passed a clinical problem to the first participant. Participants 2-5 waited outside the door. Then participant 2 entered and participant #1 passed the information to #2, then #2 to #3 and so on. The participants who are not actively involved were given specific observation tasks. A feedback round followed, discussing the observation tasks. Subsequently, the ISBAR scheme [18], which was already established at the faculty and listed in the NKLM, was discussed as a tool for systematic handover. The process was then repeated. 

For the *feedback *station, clinical vignettes described an IP conflict from a monoprofessional perspective (vignette A). A corresponding Vignette B described the same situation from the other profession’s perspective. Feedback was provided in accordance with vignette A and the recipient responded according to vignette B. This was followed by a feedback round analogous to *telephone game* station, with a repetition of the feedback rules based on the WWW scheme [19]. Further rounds followed.

At the *team communication* station, a clinical problem was communicated by a standardized patient to two participants from different professions in different ways. The end of the simulation was initiated by the necessity for exchange and agreement between the two participants, e.g. through patient re-evaluation or handover in the emergency department. A feedback round was administered, analogous to the forementioned stations. Emphasis was placed on the importance of exchange and active questioning, following the principle “share and seek” [20]. Two iterations were performed.

## Implementation in the curriculum

MS were in their first clinical year, while the NT and PT were in their second year of training.

For MS, the course was linked to an existing curricular, timetable-fixed course (clinical examination course). Some IP contents were previously discussed in medical sociology lectures and seminars (competence level 1 according to NKLM). Outside the IP course, NTs were only trained on the topic of IP information transfer in their second year of training. PT received IP topics in the context of the Team Resource Management block with 40 teaching units, taught monoprofessionally in advance.

Dates for Komm2gether and KUKIP were coordinated among the professional groups and scheduled mid-semester. Attendance was mandatory for MS and NT, and for PT only for Komm2gether. In the winter term 2023/24, the course was conducted with MS (n=168), PT(n=28) and NT (n=30).

## Evaluations

The aim of quantitative and qualitative evaluation was to adapt the course content to the participants’ needs and to assess participants’ attitudes towards the new teaching content. The evaluation was designed by the IP WG team members. Data collection and analysis were carried out digitally via QR code and the Evasys-System at the end of the course. Participants were asked about didactics, practical relevance, relevance to the participants and the commitment of the instructors. In addition to open-ended questions for qualitative analysis, quantitative data using a Likert scale (1-6; 1=I agree, 6=I disagree) was collected for the Komm2gether. At KUKIP, each station was assessed for clinical relevance and sufficiency of practice opportunities using yes/no questions. Statistical analysis was performed using SPSS, applying the unpaired T-test, the Welch T-test and the Kruskal-Wallis test. All respondents consented to scientific analysis. After reviewing the project, the local ethics committee deemed no formal ethical approval necessary. Participation in the survey was voluntary.

The feedback on the IP course was very heterogeneous. The response rates for Komm2gether were 14% for MS, 33% for NT and 25% for PA. For KUKIP, the overall response rates were 61% for the station *team communication*, 59% for *feedback* and 34% for *telephone game*, respectively.

### Evaluation of Komm2gether

In the quantitative analysis, large differences were observed in the perception of course content between professional groups (see table 3 (Tab. 3)). NT rated the topics as significantly more interesting than MS. They perceived the content as significantly better aligned with their level of knowledge compared with MS and PT and reported a significantly higher perceived knowledge gain than MS and PT. Accordingly, the overall course rating was significantly higher among NT. Notably, an extreme range of scores (1-6) was observed, reflected in the high standard deviations, especially among MS. All professional groups rated the teaching staff as friendly and open-minded. In contrast to MS, PT desired significantly more time for exchange. MS rated the teaching unit as significantly inappropriate (too long) compared to NT and PT. Some differences did not reach statistical significance due to the low response rate.

Overall, 31 free-text comments were collected on Komm2gether. The qualitative statements reflected the quantitative results. An overview of the free-text comments highlighted the relevance of the IP topics, the attendance of various professional groups, and the opportunity for exchange as particularly positive.

Overall, 31 free-text comments were collected on Komm2gether. The qualitative statements reflected the quantitative results. An overview of the free-text comments highlighted the relevance of the IP topics, the attendance of various professional groups, and the opportunity for exchange as particularly positive.

### Evaluation of KUKIP

In the quantitative analysis, a consistent pattern was observed across all three stations (see table 4 (Tab. 4)). All stations were rated as clinically relevant, and participants reported sufficient opportunities for practice.

Comments and informal feedback from the course instructors noted, that there were complaints about the unequal ratio of participants from the different professional groups.

In the qualitative evaluation, for *telephone game*, cases were criticized as partly unrealistic and too complex due to technical terminology. In *team communication*, the conflict potential was considered insufficient to increase case complexity.

During the pilot phase, participants were reluctant to take an active role in the *feedback* station. This was overcome during the course phase by asking participants to first act out a negative example. Feedback session, which during the pilot phase were only based on errors, were considered too obvious, and participant responses were correspondingly shallow. In the course, the *feedback* station was enhanced by introducing ambiguous vignettes, allowing for differing opinions, and by providing vignettes for feedback recipients that pre-structured their responses, thereby increasing situational complexity.

In the current version, despite these adjustments, even more complexity was desired, which could be achieved by using standardized patients from the HP.

## Discussion

This paper shows that the establishment of a new compulsory teaching unit on interprofessional (IP) communication with limited resources is feasible. Owing to the strong networking within the IP teaching team, it was possible to implement an IP course addressing IP roles and communication. Mandatory attendance for all participants was introduced deliberately in light of the following challenges encountered.

### Openness to IP topics

Current data indicates that introducing an obligatory IP program is demanding but worthwhile. This approach reaches not only those MS, NT, and PT who already have a strong interest in IP topics, but also those with less awareness who may benefit the most. Evaluation results show that interest in IP learning varies considerably both between and within professional groups. Therefore, when implementing IP courses, the relevance of the subject matter must be clearly communicated and illustrated through practical examples, such as best- or worst-practice videos or expert panels

Findings in the literature regarding profession-specific readiness for IP learning are inconsistent. The One Health Project by Roopnarine et al. [21] reported lower readiness among MS – measured by the Readiness for Interprofessional Learning Scale (RIPLS) – compared with students of other HP. Oliveira et al. [22] similarly found higher RIPLS scores in nursing, pharmacy, and dental students than in MS. In contrast, Song et al. [23] reported comparable readiness for IP learning between NT and MS. Personal and gender-specific differences have also been described [24].

Furthermore, a discrepancy between subjective teamwork performance and objectively assessed performance in OSCEs has been reported [25]. This misjudgement, combined with limited readiness for IP learning among some MS, may contribute to a potentially dangerous gap that could hinder effective teamwork in clinical practice. In this context, compulsory IP learning appears to be an appropriate means of establishing conditions for successful teamwork. Mandatory attendance also ensures a multiprofessional composition of the face-to-face sessions, which is a prerequisite for conducting IP courses.

### Curricular integration

When linking different educational curricula within a timetable, the difficulty of identifying shared course times inevitably arises. Scheduling sessions in off-peak times in the timetables (e.g. early evening) may improve feasibility, as may dividing the cohort and offering sessions twice, particularly for Komm2gether. In the long term, joint curriculum planning is desirable. Scheduling difficulties across disciplines are well known in the literature [26] and should therefore be considered early on. Framework conditions – such as examination periods and the clinical placements of the respective programs – should also be considered.

### Number of participants from different professional groups

In Regensburg, significantly more MS than NT and PT were enrolled, resulting in markedly imbalanced group compositions. This issue is also described elsewhere when implementing compulsory IP courses [27]. As cohort sizes cannot be altered, two solutions are conceivable:

1. involving additional professional groups, or
2. involving several institutions offering the same training programs.

### Differences in prior knowledge

Aligning course content with the heterogeneous prior knowledge of participants represents the greatest challenge. As our example shows, this point has not yet been successfully addressed. PT, who had completed Team Resource Management in their first year, found the topics interesting but felt that the level did not match their existing knowledge (Likert 4.4). The NT, on the other hand, rated the content as very well aligned with their level (Likert 1.3). The MS also perceived the content as not well adapted to their knowledge (Likert 3.6), despite having had little prior IP teaching, especially not in an active role. A mismatch between prior knowledge and course level can hinder achievement of learning objectives. Lestari et al. [28] described an IP course in Asia from which participants of certain professions emerged uncertain and unmotivated. Course content should avoid overwhelming individual groups to prevent widening professional divides.

### Different needs of learners

MS considered the teaching unit too long (see table 3 (Tab. 3), Item 5) and regarded the time for interprofessional exchange as sufficient (see table 3 (Tab. 3), Item 12), whereas PT rated the exchange time as insufficient. From the available data, one may infer PT – with considerable prior training in the topic – had higher expectations regarding informal IP exchange. A prior assessment of expectations across professional groups may help to address differing needs.

Overall, establishing an IP course involves specific challenges that do not arise in other educational settings. Close collaboration within an IP teaching team can help overcome several of these obstacles. A preliminary survey of the needs of the individual profession’s curricular integration with mandatory attendance, inclusion of as many participants from other professions as possible, analysis of prior knowledge and expectation, and early scheduling coordination support successful project implementation. During delivery, the relevance of the topic should be emphasized, opportunities for exchange should be provided, and simulations should be utilized. When expanding to additional professions, course content should be adapted and re-evaluated.

### Limitations

The explanatory power of the quantitative evaluations is limited owing to the low response rate and should be interpreted as indicative only. PT did not have mandatory attendance for KUKIP for administrative reasons, and some (likely due to upcoming exams) did not attend, resulting in some monoprofessional sessions.

## Conclusion

Close cooperation between educators from different HP enables resource-efficient implementation of a new, first compulsory IP teaching unit at an early stage of training. Given the widely acknowledged importance of IP communication for effective teamwork, similar teaching units should be established at other institutions.

Specific challenges – such as unequal participant numbers and differing needs – must be continuously monitored and can be addressed through anticipatory planning. Course content must avoid overburdening individual professions, as this may reinforce existing divides and undermine the purpose of IP education.

From our perspective, a compulsory format is essential to reach those with the greatest learning needs.

To meet the various needs of participants and ensure successful teamwork, a spiral curriculum with increasing complexity – e.g., expansion towards IP conflict communication or IP teamwork later in training – is advisable.

## Acknowledgements

We thank the “Stiftung Innovation in der Hochschullehre” for supporting the further development of the project and publication of the manuscript.

## Authors’ ORCIDs

- Lars S. Maier: [0000-0001-9915-4429]
- Jobst-Hendrik Schultz: [0000-0001-9433-3970]

## Competing interests

The authors declare that they have no competing interests. 

## Supplementary Material

Examples of case vignettes of the KUKIP stations

## Figures and Tables

**Table 1:**
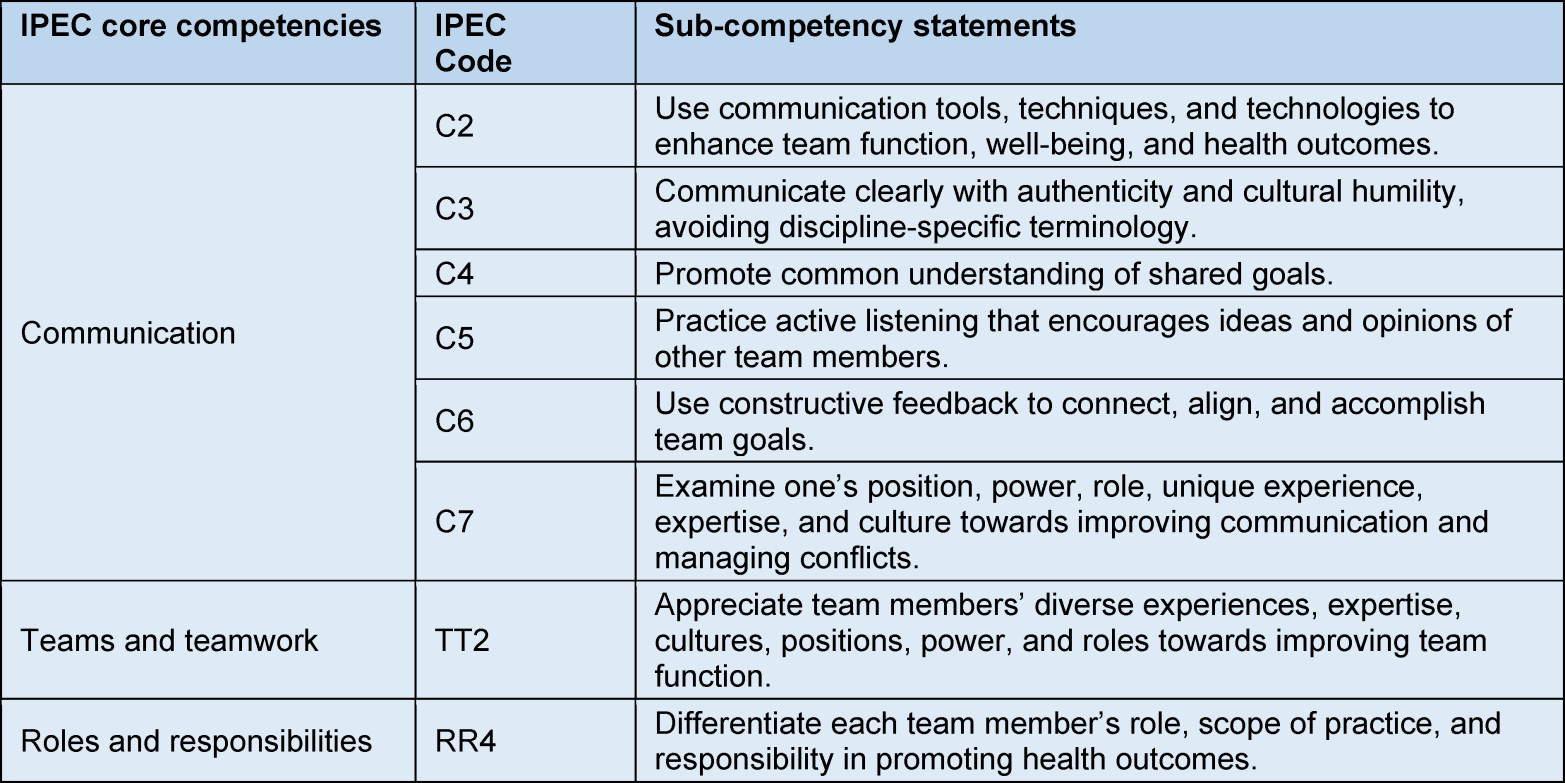
Sub-competency statements of IPEC core competencies (IP education collaborative)

**Table 2:**
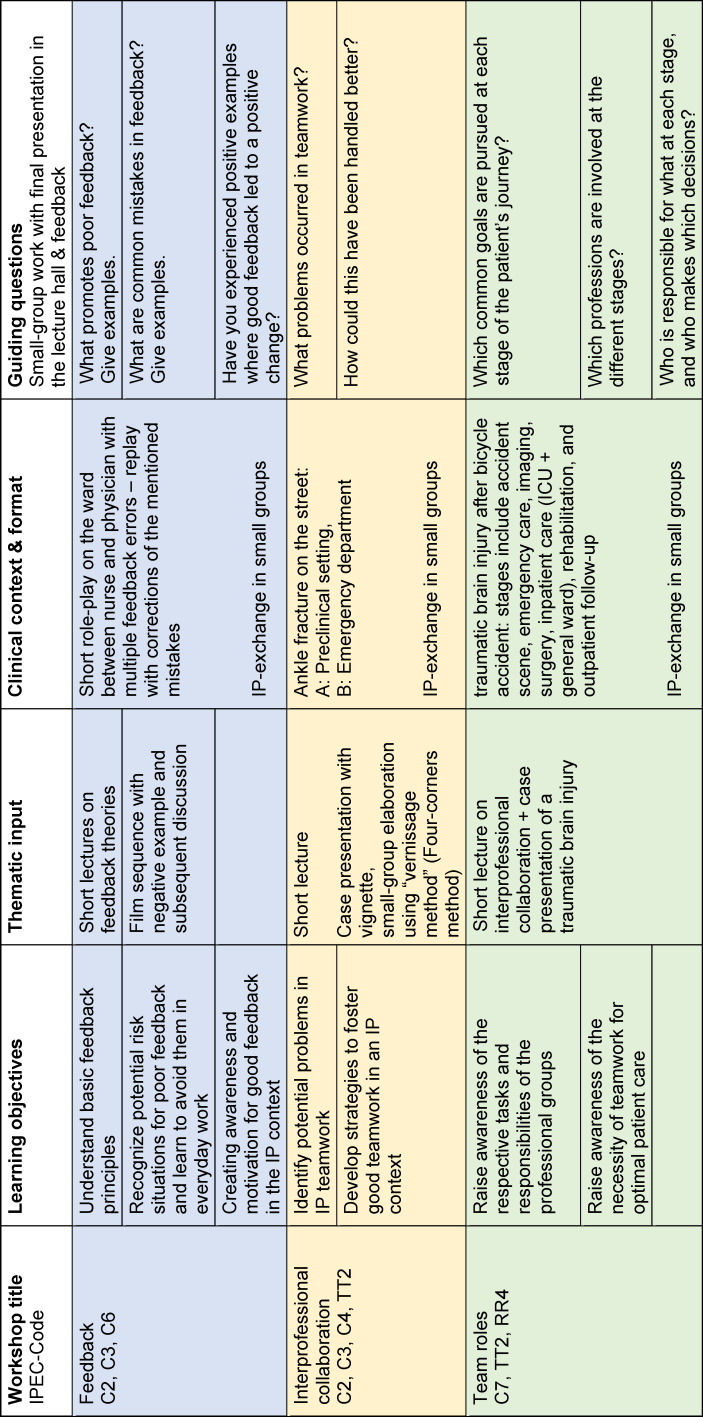
Overview of the 3 iInterprofessional (IP) workshops at Komm2gether (n=108 per session)

**Table 3:**
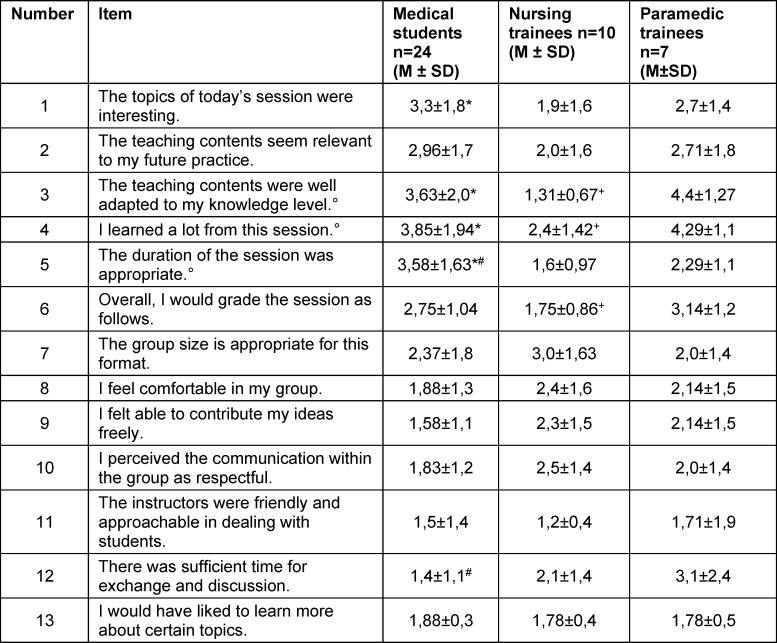
Evaluation of the Komm2gether by participants Mean values (M±SD) using a Likert scale (1-6, 1=I agree, 6=I disagree). Significant differences in two-sided Welch t-tests at p<0.05 are marked as follows: MS and NT with *, MS and PT with #, NT and PT with +. Kruskal-Wallis test p<0.05 indicated with °.

**Table 4:**
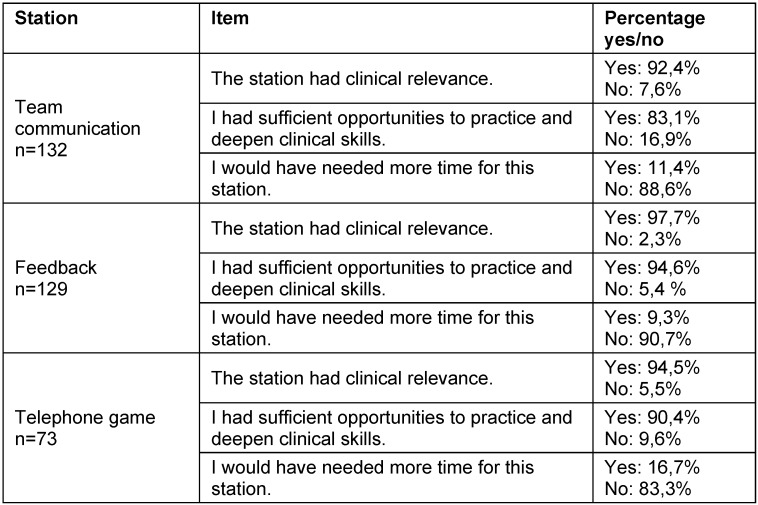
Evaluations of the KUKIP stations
