# Supplementary material for: The particularities and challenges of establishing a curricular teaching unit on interprofessional communication in healthcare
Source: GMS J Med Educ. 2026 Apr 15;43(4):Doc49. doi: 10.3205/zma001843 (PMC13124539; doi:10.3205/zma001843)
Supplement: Examples of case vignettes of the KUKIP stations [file JME-43-49-s-001.pdf]

Attachment 1: Examples of case vignettes of the KUKIP stations

**Station**	**General case vignette**	**Pre-briefing for participants**	**SP**
Team communication	• Task: Joint planning of further management for a patient with pneumonia.<br>• SP in bed, currently having breakfast.<br>• MS arrives for the ward round and discusses further management, afterwards a separate consultation with the NT.<br>• Diabetes mellitus was newly diagnosed during this hospital stay and insulin therapy was initiated. The MS and SP agree on discharge today. As always, bed shortage applies. MS leaves.<br>• impatient, insecure, easily agitated patient, strongly insisting on being discharged<br>• End: SP demands discharge documents from both the MS and the NT at the nurses' station.	For NT:<br>It is Friday.<br>You are in charge of a patient on a general ward who has been successfully treated for pneumonia. During treatment, a diabetes mellitus was diagnosed.<br>Discharge is expected soon.<br>You see the patient during your morning round.	For NT:<br>• Extremely worried due to lack of knowledge regarding insulin therapy.<br>• No personal device (glucose meter): How should she continue at home?<br>• Erythema at the left forearm at the site of the peripheral IV catheter.<br>• General practitioner currently unavailable.
		You are overseeing a patient on a general ward. She was hospitalized due to pneumonia, which has now been successfully treated with antibiotics. During the stay, a diabetes mellitus was diagnosed and therapy has been initiated.<br>Discharge is expected soon.<br>You see the patient during your ward round.	For MS:<br>• SP receives a phone call during the ward round regarding preparations for a party.<br>• Very eager to be discharged.<br>• Medications for the weekend must be provided.
Feedback	• General scenario during a night shift between MS and NT.<br>• MS not reachable by phone at night regarding a medical question (not an emergency).<br>• feedback session takes place the next morning, initiated by the NT as the feedback giver.	Sender MS (Vignette A):<br>During your last night shift, you received several phone calls from the nursing staff on the surgical ward about what you perceived as "minor issues." For example: administration of pain medication, although a PRN order was already documented; or notification of a patient who had not yet arrived. You were annoyed by this and now address the nurse the following day.	No SP used.<br><br>Role-play between two participants from different health professions.
		Receiver NT (Vignette B):<br>You, as the NT, respond that neither the pain situation nor the arrival of a patient were "minor issues." You want to discuss whether the prescribed dosage was sufficient. You state that physicians must always be reachable for such questions during night duty. This is part of the job and should be considered self-evident.	

Attachment 1 to Fredersdorf S, Maier LS, Stocker I, Staab F, Wiesner A, Fehlner C, Schultz JH, Keil S. *The particularities and challenges of establishing a curricular teaching unit on interprofessional communication in healthcare.* GMS J Med Educ. 2026;43(4):Doc49. DOI: 10.3205/zma001843

Station	General case vignette	Pre-briefing for participants	SP
Telephone game	• 65-year-old patient hospitalized due to myocardial infarction one day ago.<br>• Monitored on intermediate care unit.<br>• Unable to sleep.<br>• Severe groin pain after cardiac catheterization with stent implantation; patient did not adhere to bed rest.<br>• Mentions newly occurring mild chest discomfort.<br>• Issues to be handed over: insomnia, pain, groin issues, chest discomfort.	For Participant #1:<br>• 65-year-old patient hospitalized due to myocardial infarction one day ago.<br>• Monitoring on intermediate care unit.<br>• Unable to sleep.<br>• Severe groin pain after cardiac catheterization with stent implantation; patient did not adhere to bed rest.<br>• Mentions newly occurring mild chest discomfort.<br>• Issues to be handed over: insomnia, pain, groin issues, chest discomfort.	• “I could not sleep last night because of the groin pain after the heart catheterization. I tried to get up during the night and since then it hurts even more. I just couldn’t sleep. The beds are uncomfortable. I usually sleep on my side. Do you know body pillows for side sleepers? They’re wonderful.”<br>• “What exactly was done during the angiography? I could see something on the screen, like branches. I enjoy walking in the woods, so it reminded me of trees.”<br>• “I now feel a slight pressure on my chest again.”<br>• “Can you tell me when I can finally get up? Because of the pain in the groin.”<br>• “I am flying to Italy next week for vacation. It’s a beautiful country. We have a nice hotel, all-inclusive, directly at the beach. It was a great deal. Unfortunately, it’s non-refundable.”

Attachment 1 to Fredersdorf S, Maier LS, Stocker I, Staab F, Wiesner A, Fehlner C, Schultz JH, Keil S. *The particularities and challenges of establishing a curricular teaching unit on interprofessional communication in healthcare.* GMS J Med Educ. 2026;43(4):Doc49. DOI: 10.3205/zma001843
